# Supplementary material for: Genomic Insights on the Carbon-Negative Workhorse: Systematical Comparative Genomic Analysis on 56 Synechococcus Strains
Source: Bioengineering (Basel). 2023 Nov 18;10(11):1329. doi: 10.3390/bioengineering10111329 (PMC10669429; doi:10.3390/bioengineering10111329)
Supplement: Supplementary file 1 [file bioengineering-10-01329-s001.zip › bioengineering-2667018-supplementary.pdf]

bioengineering

MDPI

Supplementary

Genomic Insights on the Carbon-Negative Workhorse: Systematical Comparative Genomic Analysis on 56 *Synechococcus* Strains

Meiwen Qian, Xiao Han, Jiongqin Liu, Ping Xu and Fei Tao *

School of Life Sciences and Biotechnology, Shanghai Jiao Tong University, Shanghai 200240, China; 119080930114@sjtu.edu.cn (M.Q.); smile56@sjtu.edu.cn (X.H.); 697_jiongqinliu@sjtu.edu.cn (J.L.); pingxu@sjtu.edu.cn (P.X.)

* Correspondence: taofei@sjtu.edu.cn; Tel.: +86-21-34206647

Table S1. Dominant ecological conditions for *Synechococcus* clades.

Ecosystem	Clade	subcluster	Ecological conditions with maximum relative abundance	strain
Marine	5.1	Ⅰ	Nutrient open ocean waters in coastal or temperate zones are mainly above 30° north latitude	CC9311, MVIR-18-1, PROS-9-1, ROS8604
		Ⅱ	Offshore, low-nutrient tropical or subtropical waters between 20°N and 30°N	A15-62, CC9605, KORDI-52, M16.1, PROS-U-1, WH8109, A15-44, RS9902, TAK9802, KORDI-100
		Ⅳ	Nutrient open waters in coastal or temperate zones	CC9902
		V	Subtropical areas between 30° and 40° north latitude	WH7803, BMK-MC-1
		VI	upwelling zone	MEDNS5, PROS-7-1
		VII	Open oceans in the Northern Hemisphere, spanning latitudes covering tropical and subtropical regions	A15-60, A18-25c, NOUM97013
		VIII	Temperate regions with extensive salt tolerance	RS9909, WH8101
		CRD1	Iron poverty, subequatorial Pacific	BIOS-U3-1, MIT S9220
		UC-A	Tropical waters of the western North Pacific	KORDI-100
		WPC1	Large oligotrophic water body, open water	A15-127, KORDI-49
	5.2		Low nutrient Antarctic, subtropical coastal	CB0101, CBW1002, CBW1004, CBW1006, CBW1107, CBW1108, LTW-R, SynAce01, RSCCF101
	5.3		Mediterranean	MINOS11, RCC307
	C(3)		Lack of phycoerythrin, euryhaline, requires vitamin B12 for growth	NIES-970, PCC 11901, PCC 7002, PCC 7003, PCC 7117, PCC 73109, PCC 8807

Freshwater	Inland water bodies in North America, including freshwater lakes and reservoirs	PCC 6301, PCC 6312, PCC 7502, PCC 7942, UTEX 2973, UTEX 3055
Thermal springs	High temperature, near the bottom of hot springs	JA-2-3B′a(2-13), JA-3-3Ab

Table S2. Status of pan-genome comparative analysis.

Strain	Core genes	Accessory genes	Unique genes	Exclusively absent genes
A15-44	275	2292	112	0
A15-60	275	2230	87	0
A15-62	275	2092	80	0
A15-127	275	2154	147	1
A18-25c	275	2225	52	0
BIOS-U3-1	275	2181	280	0
BMK-MC-1	275	2325	109	0
CB0101	275	2032	474	1
CBW1002	275	2675	153	0
CBW1004	275	2115	704	4
CBW1006	275	2725	219	0
CBW1107	275	2173	506	0
CBW1108	275	2095	593	2
CC9311	275	2112	225	0
CC9605	275	2078	197	0
CC9902	275	1832	232	0
JA-2-3B′a(2-13)	275	2091	223	2
JA-3-3Ab	275	2068	145	3
KORDI-49	275	2160	125	1
KORDI-52	275	2130	201	0
KORDI-100	275	2022	506	0
LTW-R	275	1814	400	0
M16.1	275	1945	42	0
MEDNS5	275	2106	122	0
MINOS11	275	2007	188	0
MIT S9220	275	2099	164	0
MVIR-18-1	275	2087	123	0
NIES-970	275	2326	206	0
NOUM97013	275	2202	134	0
PCC 6301	275	2296	3	5
PCC 6312	275	1245	1896	3
PCC 7002	275	2717	102	0
PCC 7003	275	2566	188	0
PCC 7117	275	2699	148	0
PCC 7502	275	1060	1904	6
PCC 7942	275	2376	4	0
PCC 8807	275	2643	122	0
PCC 7942	275	2622	108	1
PCC 73109	275	2630	93	0
PROS-7-1	275	2229	128	0

PROS-9-1	275	1894	127	0
PROS-U-1	275	2175	187	0
RCC307	275	1997	86	1
ROS8604	275	2265	276	2
RS9902	275	2126	124	0
RS9907	275	2275	326	1
RS9909	275	2183	125	3
RSCCF101	275	1593	753	8
SYN20	275	2135	295	1
SynAce01	275	1919	443	3
TAK9802	275	1968	78	0
UTEX 2973	275	2366	7	0
UTEX 3055	275	2276	255	0
WH 7803	275	2074	73	0
WH 8101	275	2188	145	1
WH 8109	275	1882	78	0

Table S3. Gene families association with thermal adaptation.

KO	Gene name	Path ID	Pathway
K00016	LDH, ldh	ko00270	Cysteine and methionine metabolism
K01008	selD, SEPHS	ko00450	Selenocompound metabolism
K00137	prr	ko00330	Arginine and proline metabolism
K00276	AOC2, tynA	ko00260	Glycine, serine and threonine metabolism
K00549	metE	ko00270	Cysteine and methionine metabolism
K00865	glxK	ko00260	Glycine, serine and threonine metabolism
K01243	mtnN, mtn,	ko00270	Cysteine and methionine metabolism
K01473	hyuA	ko00330	Arginine and proline metabolism
K01474	hyuB	ko00330	Arginine and proline metabolism
K01485	codA	ko00330	Arginine and proline metabolism
K04072	adhE	ko00350	Tyrosine metabolism
K05825	LYSN	ko00300	Lysine biosynthesis
K12339	cysM	ko00270	Cysteine and methionine metabolism
K00622	nat	ko05204	Human Diseases
K11594	DDX3X, bel	ko05203	Human Diseases
K00030	IDH3	ko00020	Citrate cycle (TCA cycle)
K00656	pflD	ko00620	Pyruvate metabolism
K00852	rbsK, RBKS	ko00030	Pentose phosphate pathway
K00925	ackA	ko00620	Pyruvate metabolism
K01236	treZ, glgZ	ko00500	Starch and sucrose metabolism
K01512	acyP	ko00620	Pyruvate metabolism
K01621	xfp, xpk	ko00030	Pentose phosphate pathway
K06044	treY, glgY	ko00500	Starch and sucrose metabolism
K06623	CDKN2D, P19	ko04110	Cell cycle
K10352	MYH	ko04530	Tight junction
K00299	ssuE	ko00920	Sulfur metabolism
K02586	nifD	ko00910	Nitrogen metabolism
K02588	nifH	ko00910	Nitrogen metabolism
K02591	nifK	ko00910	Nitrogen metabolism
K03666	hfq	ko03018	RNA degradation

K09517	DNAJB11	ko04141	Protein processing in endoplasmic reticulum
K09540	SEC63	ko03060	Protein export
K13525	VCP, CDC48	ko04141	Protein processing in endoplasmic reticulum
K02843	waaF, rfaF	ko00540	Lipopolysaccharide biosynthesis
K14048	ureAB	ko05120	Infectious diseases
K02006	cbiO	ko02010	ABC transporters
K02007	cbiM	ko02010	ABC transporters
K02008	cbiQ	ko02010	ABC transporters
K02045	cysA	ko02010	ABC transporters
K02046	cysU	ko02010	ABC transporters
K02047	cysW	ko02010	ABC transporters
K02048	cysP, sbp	ko02010	ABC transporters
K02424	fliY	ko02010	ABC transporters
K05813	ugpB	ko02010	ABC transporters
K05814	ugpA	ko02010	ABC transporters
K05815	ugpE	ko02010	ABC transporters
K05845	opuC	ko02010	ABC transporters
K05846	opuBD	ko02010	ABC transporters
K05847	opuA	ko02010	ABC transporters
K09811	ftsX	ko02010	ABC transporters
K09812	ftsE	ko02010	ABC transporters
K09970	aapQ, bztB	ko02010	ABC transporters
K09971	aapM, bztC	ko02010	ABC transporters
K10009	ABC.CYST.P	ko02010	ABC transporters
K11069	potD	ko02010	ABC transporters
K11070	potC	ko02010	ABC transporters
K11071	potB	ko02010	ABC transporters
K11072	potA	ko02010	ABC transporters
K11707	troA, mntA	ko02010	ABC transporters
K11710	troB, mntB	ko02010	ABC transporters
K11951	cmpB	ko02010	ABC transporters
K11952	cmpC	ko02010	ABC transporters
K13892	gsiA	ko02010	ABC transporters
K15576	nrtA, cynA	ko02010	ABC transporters
K16201	dppC1	ko02010	ABC transporters
K16915	cbiL	ko02010	ABC transporters
K00077	panE, apbA	ko00770	Metabolism of cofactors and vitamins
K00231	PPOX, hemY	ko00860	Metabolism of cofactors and vitamins
K01239	iunH	ko00760	Metabolism of cofactors and vitamins
K03153	thiO	ko00730	Metabolism of cofactors and vitamins
K03707	tenA	ko00730	Metabolism of cofactors and vitamins
K05895	cobK-cbiJ	ko00860	Metabolism of cofactors and vitamins
K18278	THI5	ko00730	Metabolism of cofactors and vitamins
K10396	KIF5	ko04728	Dopaminergic synapse
K00087	ygeS, xdhA	ko00230	Purine metabolism
K01518	NUDT2	ko00230	Purine metabolism
K03652	MPG	ko03410	Base excision repair
K00425	cydA	ko02020	Two-component system
K00426	cydB	ko02020	Two-component system
K01077	phoA, phoB	ko02020	Two-component system
K07670	mtrA	ko02020	Two-component system

K11328	nrsS, rppB	ko02020	Two-component system
K11525	pixJ	ko02020	Two-component system
K18649	IMPL2	ko04070	Phosphatidylinositol signaling system

Table S4. Gene families association with cold adaptation.

KO	Gene name	Path ID	Pathway
K00042	garR, glxR	ko00630	Valine, leucine and isoleucine degradation
K00067	rfbD, rmlD	ko00521	Alanine, aspartate and glutamate metabolism
K00128	E1.2.1.3	ko00280	Histidine metabolism
K00383	GSR, gor	ko00480	Histidine metabolism
K00678	LRAT	ko00830	Histidine metabolism
K10238	thuG, sugB	ko02010	Glutathione metabolism
K11927	rhlE	ko03018	Taurine and hypotaurine metabolism
K00833	bioA	ko00780	Streptomycin biosynthesis
K00864	glpK, GK	ko00561	Streptomycin biosynthesis
K00973	rfbA, rffH	ko00521	Streptomycin biosynthesis
K01437	ASPA, aspA	ko00250	Streptomycin biosynthesis
K01594	CSAD	ko00430	Glyoxylate and dicarboxylate metabolism
K01623	ALDO	ko00010	Glycolysis / Gluconeogenesis
K01663	HIS7	ko00340	Starch and sucrose metabolism
K01679	fumC	ko05200	Amino sugar and nucleotide sugar metabolism
K01710	rfbB, rffG	ko00521	Starch and sucrose metabolism
K01790	rfbC, rmlC	ko00521	Protein processing in endoplasmic reticulum
K02438	treX, glgX	ko00500	RNA degradation
K02564	nagB	ko00520	Cancers
K03465	thyX, thy1	ko00670	Drug resistance
K06443	lcyB, crtY	ko00906	Drug resistance
K07190	PHKA	ko04020	Glycerolipid metabolism
K07264	arnT, pmrK	ko01503	ABC transporters
K07659	ompR	ko02020	ABC transporters
K08218	ampG	ko01501	ABC transporters
K08679	E5.1.3.6	ko00500	Metabolism of cofactors and vitamins
K09523	DNAJC3	ko04141	Metabolism of cofactors and vitamins
K10237	thuF, sugA	ko02010	Metabolism of cofactors and vitamins
K13342	PEX5, PXR1	ko04146	Metabolism of terpenoids and polyketides
K18104	abcA, bmrA	ko02010	Calcium signaling pathway
K18911	egtD	ko00340	Two-component system
K18912	egtB	ko00340	Peroxisome

Table S5. Gene families association with halotolerance.

KO	Gene name	Path ID	Pathway
K00020	mmsB	ko00280	Valine, leucine and isoleucine degradation
K00031	IDH1, IDH2, icd	ko04146	Valine, leucine and isoleucine degradation
K00042	garR, glxR	ko00630	Valine, leucine and isoleucine degradation
K00067	rfbD, rmlD	ko00521	Arginine and proline metabolism
K00111	glpA, glpD	ko00564	Glycine, serine and threonine metabolism
K00128	E1.2.1.3	ko00280	Cysteine and methionine metabolism
K00263	E1.4.1.9	ko00280	Cysteine and methionine metabolism
K00286	proC	ko00330	Arginine and proline metabolism
K00390	cysH	ko00920	Arginine and proline metabolism

K00432	E1.11.1.9	ko00590	Arginine and proline metabolism
K00517	E1.14.-.-	ko00945	Cysteine and methionine metabolism
K00605	gcvT, AMT	ko00260	Histidine metabolism
K00651	metA	ko00270	Valine, leucine and isoleucine biosynthesis
K00652	bioF	ko00780	Cysteine and methionine metabolism
K00678	LRAT	ko00830	Glycine, serine and threonine metabolism
K00681	ggt	ko00430	Glycine, serine and threonine metabolism
K00690	E2.4.1.7	ko00500	Phenylalanine, tyrosine and tryptophan biosynthesis
K00748	lpxB	ko00540	Histidine metabolism
K00759	APRT, apt	ko00230	Cysteine and methionine metabolism
K00784	rnz	ko03013	Cysteine and methionine metabolism
K00791	miaA, TRIT1	ko00908	Histidine metabolism
K07462	recJ	ko03410	Taurine and hypotaurine metabolism
K08289	purT	ko00670	Selenocompound metabolism
K08303	K08303	ko05120	Taurine and hypotaurine metabolism
K00793	ribE, RIB5	ko00740	Streptomycin biosynthesis
K00797	speE, SRM	ko00270	Stilbenoid, diarylheptanoid and gingerol biosynthesis
K00830	AGXT	ko04146	Glyoxylate and dicarboxylate metabolism
K00847	scrK	ko00051	Starch and sucrose metabolism
K00859	coaE	ko00770	Fructose and mannose metabolism
K00860	cysC	ko00920	Fructose and mannose metabolism
K00864	glpK, GK	ko00561	Pyruvate metabolism
K00919	ispE	ko00900	Pyruvate metabolism
K00939	adk, AK	ko00230	Starch and sucrose metabolism
K00943	tmk, DTYMK	ko00240	Amino sugar and nucleotide sugar metabolism
K00946	thiL	ko00730	Starch and sucrose metabolism
K00950	folK	ko00790	Amino sugar and nucleotide sugar metabolism
00969	nadD	ko00760	Fructose and mannose metabolism
K00971	manC, cpsB	ko00051	Starch and sucrose metabolism
K00974	cca	ko03013	Glyoxylate and dicarboxylate metabolism
K01008	selD, SEPHS	ko00450	Cell growth and death
K01012	bioB	ko00780	Drug resistance
K01046	E3.1.1.3	ko00561	Drug resistance
K01082	cysQ, BPNT1	ko00920	Drug resistance
K01191	E3.2.1.24	ko00511	Drug resistance
K01448	amiA, amiB, amiC	ko01503	Sulfur metabolism
K01470	E3.5.2.10	ko00330	Sulfur metabolism
K01480	speB	ko00330	Sulfur metabolism
K01485	codA	ko00330	Nitrogen metabolism
K01512	acyP	ko00620	Photosynthesis - antenna proteins
K01589	purK	ko00230	Oxidative phosphorylation
K01591	pyrF	ko00240	Photosynthesis - antenna proteins
K01594	CSAD	ko00430	Nitrogen metabolism
K01611	speD, AMD1	ko00270	Photosynthesis
K01627	kdsA	ko00540	Photosynthesis
K01663	HIS7	ko00340	Photosynthesis
K01665	pabB	ko00790	Photosynthesis
K01687	ilvD	ko00290	Photosynthesis
K01725	cynS	ko00910	Photosynthesis
K01739	metB	ko00270	Oxidative phosphorylation
K01754	ilvA, tdcB	ko00260	Environmental adaptation

K01758	CTH	ko00260	Sulfur relay system
K01759	GLO1, gloA	ko04011	Sulfur relay system
K01817	trpF	ko00400	Sulfur relay system
K01897	ACSL, fadD	ko04146	Sulfur relay system
K01935	bioD	ko00780	RNA degradation
K02038	pstA	ko02010	RNA degradation
K02041	phnC	ko02010	Folding, sorting and degradation
K02092	apcA	ko00196	RNA degradation
K02160	accB, bccP	ko00620	Protein processing in endoplasmic reticulum
K02188	cbiD	ko00860	Lipopolysaccharide biosynthesis
K02224	cobB-cbiA	ko00860	Other glycan degradation
K02259	COX15	ko02020	Lipopolysaccharide biosynthesis
K02275	coxB	ko00190	Lipopolysaccharide biosynthesis
K02290	cpcG	ko00196	Lipopolysaccharide biosynthesis
K02438	treX, glgX	ko00500	Peptidoglycan biosynthesis
K02471	yddA	ko02010	Peptidoglycan biosynthesis
K02502	hisZ	ko00340	Infectious diseases
K02536	lpxD	ko00540	Glycerophospholipid metabolism
K02548	menA	ko00130	Arachidonic acid metabolism
K02549	menC	ko00130	Glycerolipid metabolism
K02552	menF	ko00130	Glycerolipid metabolism
K02564	nagB, GNPDA	ko00520	Glycerolipid metabolism
K02575	narK, nrtP, nasA	ko00910	Glycerolipid metabolism
K02638	petE	ko00195	ABC transporters
K02698	psaK	ko00195	ABC transporters
K02699	psaL	ko00195	ABC transporters
K02717	psbP	ko00195	Phosphotransferase system (PTS)
K02719	psbU	ko00195	ABC transporters
K02722	psbX	ko00195	ABC transporters
K02757	bglF	ko02060	ABC transporters
K02891	RP-L22e, RPL22	ko03010	ABC transporters
K03046	rpoC	ko03020	ABC transporters
K03060	rpoZ	ko03020	ABC transporters
K03113	EIF1, SUI1	ko03013	ABC transporters
K03270	kdsC	ko00540	ABC transporters
K03394	cobI-cbiL	ko00860	ABC transporters
K03465	thyX, thy1	ko00670	ABC transporters
K03469	rnhA, RNASEH1	ko03030	ABC transporters
K03470	rnhB	ko03030	ABC transporters
K03523	bioY	ko02010	Biotin metabolism
K03584	recO	ko03440	Retinol metabolism
K03587	ftsI	ko01501	Riboflavin metabolism
K03588	ftsW, spoVE	ko04112	Pantothenate and CoA biosynthesis
K03601	xseA	ko03430	Thiamine metabolism
K03635	MOCS2, moaE	ko04122	Folate biosynthesis
K03636	moaD	ko04122	Nicotinate and nicotinamide metabolism
K03637	moaC	ko04122	Biotin metabolism
K03639	MOCS1, moaA	ko04122	Folate biosynthesis
K03654	recQ	ko03018	Biotin metabolism
K03795	cbiX	ko00860	Porphyrin and chlorophyll metabolism
K04565	SOD1	ko04146	Porphyrin and chlorophyll metabolism

K05275	E1.1.1.65	ko00750	Ubiquinone and other terpenoid-quinone biosynthesis
K05343	treS	ko00500	Ubiquinone and other terpenoid-quinone biosynthesis
K05366	mrcA	ko01501	Ubiquinone and other terpenoid-quinone biosynthesis
K05585	ndhN	ko00190	Porphyrin and chlorophyll metabolism
K05592	deaD, cshA	ko03018	One carbon pool by folate
K06118	SQD1, sqdB	ko00520	Porphyrin and chlorophyll metabolism
K06153	bacA	ko00550	Vitamin B6 metabolism
K06444	lcyE, crtL2	ko00906	Folate biosynthesis
K06920	queC	ko00790	Metabolism of cofactors and vitamins
K07026	E3.1.3.70	ko00051	Metabolism of cofactors and vitamins
K07190	PHKA	ko04020	Metabolism of cofactors and vitamins
K07259	dacB	ko00550	Metabolism of cofactors and vitamins
K07260	vanY	ko02020	Ubiquinone and other terpenoid-quinone biosynthesis
K08479	sasA	ko02020	Zeatin biosynthesis
K08591	plsY	ko00561	Terpenoid backbone biosynthesis
K08679	E5.1.3.6	ko00500	Carotenoid biosynthesis
K08964	mtnB	ko00270	Carotenoid biosynthesis
K08967	mtnD, mtnZ, ADI1	ko00270	Purine metabolism
K09457	queF	ko00790	Purine metabolism
K09503	DNAJA2	ko04141	Pyrimidine metabolism
K09835	crtISO, crtH	ko00906	Purine metabolism
K09970	aapQ, bztB	ko02010	Pyrimidine metabolism
K09971	aapM, bztC	ko02010	DNA replication
K10111	malK, mtlK, thuK	ko02010	DNA replication
K10236	thuE	ko02010	Homologous recombination
K10237	thuF, sugA	ko02010	Mismatch repair
K10238	thuG, sugB	ko02010	Base excision repair
K10747	LIG1	ko03030	DNA replication
K11472	glcE	ko00630	MAPK signaling pathway - yeast
K11601	mntC	ko02010	Two-component system
K11602	mntB	ko02010	Calcium signaling pathway
K11603	mntA	ko02010	Two-component system
K11705	mtsB	ko02010	Two-component system
K11720	lptG	ko02010	RNA polymerase
K11754	folC	ko00790	RNA polymerase
K12119	CRY2	ko04712	RNA transport
K12573	rnr, vacB	ko03018	RNA transport
K13541	cbiGH-cobJ	ko00860	Ribosome
K13993	HSP20	ko04141	RNA transport
K17836	penP	ko01501	Peroxisome
K18534	K18534	ko00130	Peroxisome
K18912	egtB	ko00340	Peroxisome
K19003	mgdA	ko00561	Peroxisome

Table S6. Gene families association with freshwater adaptation.

KO	Gene name	Path ID	Pathway
K00027	sfcA, maeA	ko02020	TPeroxisome
K00134	GAPDH, gapA	ko04066	Transport and catabolismPeroxisome
K00259	ald	ko00250	Two-component system
K00425	cydA	ko02020	HIF-1 signaling pathway

K00426	cydB	ko02020	Two-component system
K00441	frhB	ko00680	Two-component system
K00641	metX	ko00270	Two-component system
K00764	purF, PPAT	ko00250	Two-component system
K00835	avtA	ko00290	Two-component system
K00941	thiD	ko00730	ABC transporters
K01011	tst, mpst, sseA	ko04122	ABC transporters
K01061	E3.1.1.45	ko00361	ABC transporters
K01546	kdpA	ko02020	ABC transporters
K01547	kdpB	ko02020	ABC transporters
K01548	kdpC	ko02020	ABC transporters
K01569	oxdD	ko00630	ABC transporters
K01621	xfp, xpk	ko00030	ABC transporters
K01673	cynT, can	ko00910	ABC transporters
K01711	gmd, GMDS	ko00051	ABC transporters
K01770	ispF	ko00900	Two-component system
K01809	manA, MPI	ko00051	AMPK signaling pathway
K01823	idi, IDI	ko00900	Two-component system
K01893	NARS, asnS	ko00970	Two-component system
K01995	livG	ko02010	Two-component system
K01996	livF	ko02010	Two-component system
K01997	livH	ko02010	Two-component system
K01998	livM	ko02010	Two-component system
K02012	afuA, fbpA	ko02010	Two-component system
K02040	pstS	ko02010	ABC transporters
K02045	cysA	ko02010	ABC transporters
K02046	cysU	ko02010	ABC transporters
K02047	cysW	ko02010	ABC transporters
K02048	cysP, sbp	ko02010	ABC transporters
K02191	cbiT	ko00860	ABC transporters
K02495	hemN, hemZ	ko00860	ABC transporters
K02501	hisH	ko00340	ABC transporters
K02658	pilH	ko02020	Sulfur relay system
K03272	gmhC, hldE,	ko00540	Aminoacyl-tRNA biosynthesis
K03462	NAMPT	ko00760	Replication and repair
K03550	ruvA	ko03440	Alanine, aspartate and glutamate metabolism
K03778	ldhA	ko00620	Methane metabolism
K03781	katE, CAT, catB,	ko04146	Cysteine and methionine metabolism
K03841	fbp	ko04152	Alanine, aspartate and glutamate metabolism
K04564	SOD2	ko04146	Valine, leucine and isoleucine biosynthesis
K07646	kdpD	ko02020	Thiamine metabolism
K07657	phoB	ko02020	Xenobiotics biodegradation and metabolism
K08641	vanX	ko02020	Glyoxylate and dicarboxylate metabolism
K11520	manS	ko02020	Pentose phosphate pathway
K11521	K11521, manR	ko02020	Nitrogen metabolism
K11522	pixG	ko02020	Fructose and mannose metabolism
K11526	pixL	ko02020	Terpenoid backbone biosynthesis
K11780	cofG	ko00680	Fructose and mannose metabolism
K11781	cofH	ko00680	erpenoid backbone biosynthesis
K11950	cmpA	ko02010	Porphyrin and chlorophyll metabolism
K11951	cmpB	ko02010	Porphyrin and chlorophyll metabolism

K11952	cmpC	ko02010	Histidine metabolism
K11956	natD	ko02010	Lipopolysaccharide biosynthesis
K11957	natA	ko02010	Nicotinate and nicotinamide metabolism
K11958	natE	ko02010	Pyruvate metabolism
K13066	COMT	ko00940	Methane metabolism
K13522	K13522, nadM	ko00760	Methane metabolism
K13540	cobIJ	ko00860	Biosynthesis of other secondary metabolites
K13896	yejF	ko02010	Metabolism of cofactors and vitamins
K15578	nrtC, nasD	ko02010	Metabolism of cofactors and vitamins
K16881	K16881	ko00051	Fructose and mannose metabolism
